# Correspondence

**Published:** 1881-05

**Authors:** 


CORRESPONDENCE.—We are in receipt of a communication from Dr. George Reuling, containing certain statements from a Mr. Seifert in regard to the Chisolm-Reuling embroglio of last year. But as the case has been adjudicated by the State Medical Society, and a report of the proceedings connected with it is in the hands of the publication committee and will appear soon in its book of transactions for the year, we can conceive of no advantage that its publication in this journal could produce at the present time. Certainly none, unless the case were opened *de novo.*

